# Correlation of Histopathological Parameters With Molecular Markers in Carcinoma Endometrium

**DOI:** 10.7759/cureus.106256

**Published:** 2026-04-01

**Authors:** Kanika B Modi, Anjali Chandra, Aparna Dhar, Shefali Sardana, Sandeep Batra, Atul Sharma, Charu Garg, Harit Chaturvedi

**Affiliations:** 1 Gynaecologic and Surgical Oncology, Max Super Speciality Hospital, Saket, New Delhi, IND; 2 Molecular Oncology and Cancer Genetics, Max Super Speciality Hospital, Saket, New Delhi, IND; 3 Medical Oncology, Max Super Speciality Hospital, Saket, New Delhi, IND; 4 Radiation Oncology, Max Super Speciality Hospital, Saket, New Delhi, IND; 5 Surgical Oncology, Max Super Speciality Hospital, Saket, New Delhi, IND

**Keywords:** endometrial cancer, mmr-deficiency, p53-mutations, pole-mutations, promise classification

## Abstract

Background: To assess the association between molecular markers and histopathological parameters in endometrial carcinoma (EC).

Methods: This retrospective study was conducted at the Max Institute of Cancer Care, New Delhi, India, from January 2022 to May 2025, and included a total of 160 patients with EC. Molecular subtypes were determined using immunohistochemistry (IHC) for p53, mismatch repair (MMR) proteins, and POLE mutation status, and classified as "no specific molecular profile" (NSMP) where applicable. Histopathological variables, including histological subtype, tumor grade, stage, depth of myometrial invasion, lymphovascular space invasion (LVSI), and lymph node status, were evaluated for association with molecular subtypes using chi-square statistical analysis.

Results: Significant associations were observed between molecular subtypes and both histological type and LVSI. A strong correlation was noted between histopathological parameters and tumor grade (p<0.0001). However, no significant association was found between molecular subtypes and disease stage (p=0.136). POLE mutations were rare, identified in only 0.6% of cases.

Conclusions: Immunohistochemical markers based on The Cancer Genome Atlas (TCGA) classification show a strong correlation with established histopathological risk factors. Integrating molecular profiling into the routine diagnostic workup may improve prognostication and support personalized treatment strategies in EC.

## Introduction

EC represents nearly 5% of global cancer cases and 2% of cancer-related deaths among women [1]. In 2020, over 400,000 new cases were reported globally, and the number is projected to rise substantially in the coming years. In India, EC ranks as the third most prevalent gynecologic cancer, following cervical and ovarian cancers [2]. Traditionally, classification has been based on histopathological subtype, tumor grade, and stage. However, these parameters have limitations in terms of reproducibility and prognostic accuracy. Doppler ultrasound has emerged as a valuable non-invasive adjunct in the detection of EC, as studies show that malignant endometrial lesions demonstrate characteristic vascular patterns and significantly lower uterine artery Doppler indices such as resistance index and pulsatility index, thereby helping differentiate malignant from benign endometrial pathology and improving diagnostic accuracy when combined with B-mode ultrasound [3,4].

In 2013, the TCGA project revolutionized the classification of EC by introducing a molecular-based framework. Through comprehensive genomic integration analysis, EC was categorized into four distinct molecular subtypes: POLE mutated (POLE-mt), MMR-deficient (MMR-d), p53-abnormal (p53-abn), and "no specific molecular profile" (NSMP). Each subtype is characterized by unique genomic features and is associated with distinct prognostic outcomes [5]. In 2020, the European Society of Gynaecological Oncology (ESGO), the European Society for Radiotherapy and Oncology (ESTRO), and the European Society of Pathology (ESP) jointly updated the clinical guidelines for the management of EC. These revised guidelines incorporated both traditional histopathological features and emerging molecular classifications to improve prognostic accuracy and guide postoperative treatment strategies [6]. Consequently, the integration of molecular classification has become essential for more precise prognostication and individualized disease management.

The TCGA classification provides a comprehensive framework for EC using next-generation sequencing (NGS) technology. However, the widespread implementation of this approach is limited by high costs and restricted accessibility. To address these challenges, alternative methods such as the Proactive Molecular Risk Classifier for Endometrial Cancer (ProMisE) classification have been developed [7]. The ProMisE classification primarily relies on IHC, providing results comparable to TCGA, while remaining cost-effective and suitable for routine clinical use due to its quick turnaround time. The ProMisE algorithm integrates POLE mutation analysis with IHC for MMR proteins (MLH1, MSH2, MSH6, PMS2) and p53 to classify EC into four molecular subtypes [8]. The ProMisE algorithm classifies EC into four prognostic subtypes: POLE-mt, MMR-d, p53-abn, and p53-wild-type (p53-wt). Now part of the National Comprehensive Cancer Network (NCCN) and the World Health Organization (WHO) guidelines, this algorithm aids clinical decision-making, though it may not identify a small subset of copy number-high (CN-H) tumors without TP53 mutations [9]. Raffone et al. demonstrated that IHC analysis of four MMR proteins achieves high diagnostic accuracy, with a sensitivity of 0.96 and specificity of 0.95 [10]. Furthermore, they confirmed that p53 IHC is a reliable surrogate for p53 sequencing, effectively identifying p53 mutations in EC cases [11]. Due to their unique molecular profiles, histological characteristics, and prognostic outcomes, the ProMisE subgroups may reflect distinct disease entities within EC [12]. These variations point to different underlying mechanisms, risk factors, and patient presentations. Identifying specific clinical features in each subgroup could enable personalized prevention and treatment approaches in line with precision medicine [13].

Furthermore, factors such as younger age, early FIGO stage, and the use of adjuvant therapy may also impact prognosis within each group. In our study, we adopted the same molecular classification framework, which offers valuable insights into tumor biology and clinical outcomes. POLE-mt tumors are associated with an excellent prognosis, while p53-abn tumors, typically serous or high-grade endometrioids, exhibit aggressive behavior. MMR-d tumors, often linked to endometrioid histology and Lynch syndrome, demonstrate an intermediate prognosis. Tumors classified as NSMP represent the largest subgroup but display heterogeneous clinical behavior. The study evaluated the association between these molecular subtypes and key histopathological parameters in a retrospective patient cohort.

## Materials and methods

Patient selection and eligibility criteria

This observational retrospective study was conducted at the Max Institute of Cancer Care, Saket, New Delhi, India, from January 2022 to May 2025. Patients with a preoperative diagnosis of EC confined to the uterus were considered for inclusion. The diagnosis was established using hysteroscopy-guided endometrial biopsy. Disease confined to the uterus was defined preoperatively based primarily on pelvic MRI, with additional cross-sectional imaging where clinically indicated, demonstrating no radiologic evidence of extra-uterine extension, adnexal or peritoneal disease, bulky nodal disease, or distant metastasis. Contrast-enhanced computed tomography (CECT) of the chest and abdomen was performed in cases where extra-uterine disease was suspected.

**Figure 1:**
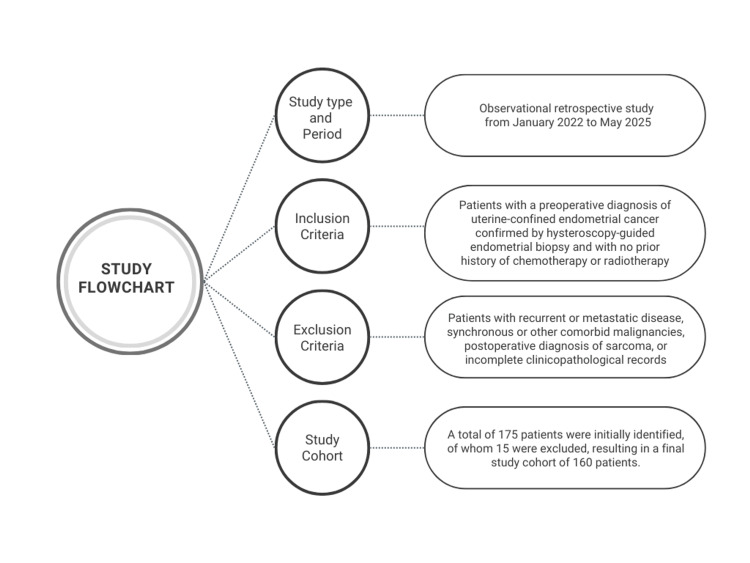
Study Flowchart

Eligible patients were those who had no prior radiotherapy, chemotherapy, or other treatments before surgery, had complete medical records, and underwent surgical treatment at our institution. Patients with recurrent or metastatic tumors, synchronous malignancies, comorbid malignancies of other tumor types, postoperative diagnoses of sarcoma, or incomplete clinicopathological records were excluded. A total of 175 patients were initially identified, of whom 15 were excluded (one patient diagnosed with endometrial stromal sarcoma and fourteen with incomplete medical records), resulting in a final study cohort of 160 patients. The study flowchart is presented in Figure 1.

Preoperative tumor grading was determined using hysteroscopy-guided biopsy, which informed the initial clinical assessment. However, final tumor grade, stage, and other pathological parameters used for analysis were based on the definitive histopathological examination of the surgical specimen. All cases were staged according to the FIGO 2023 staging system based on operative findings and final histopathology [14]. For cases managed earlier in the study period, staging was retrospectively assigned using complete clinicopathological records. Sentinel lymph node (SLN) mapping was performed in low- and intermediate-risk patients, where feasible, following the SLN algorithm. Indocyanine green dye (0.5 mg/mL) was used in accordance with NCCN recommendations, and mapped sentinel nodes were subjected to ultrastaging. Systematic pelvic ± para-aortic lymphadenectomy was performed in high-intermediate and high-risk patients or when clinically indicated. Cases in which nodal assessment was not performed, or no lymph nodes were retrieved, were recorded as “Nx/not assessed” and analyzed as a separate category. Substantial LVSI was defined as involvement of five or more lymphovascular spaces.

Data on demographics, histological subtype, tumor grade, FIGO stage, depth of myometrial invasion, LVSI, lymph node involvement, and molecular markers were extracted from institutional records and entered into a structured Microsoft Excel database (Microsoft Corp., Redmond, WA).

Molecular classification

Patients were categorized into four groups based on molecular classification: POLE-mt, MMR-d, p53-abnormal (p53-abn), and NSMP. MMR deficiency (PMS-2, MSH-6, MLH-1, MSH-2) as well as p53 were evaluated using IHC on selected formalin-fixed, paraffin-embedded (FFPE) tissue blocks according to institutional laboratory protocols. Sequence analysis of the POLE gene (exons 9 and 13) was performed using the Sanger sequencing method. POLE sequencing was limited to exons 9 and 13 to prioritize common hotspot mutations within the exonuclease domain, balancing feasibility and resource constraints in a real-world setting. However, rarer pathogenic variants outside these exons may not be detected, potentially underestimating the true POLE-mut frequency compared with sequencing of the full exonuclease domain.

All patients underwent IHC and molecular testing, and results were documented. Cases without any detectable mutations were classified under the NSMP group. A p53 mutation was defined by either diffuse, intense nuclear positivity or complete absence of nuclear staining on IHC. MMR-d was diagnosed by the loss of nuclear expression of any one of the MMR proteins. The POLE mutation was confirmed through sequencing analysis. All cases were stratified post hoc using ESGO-ESTRO-ESP 2020 risk groups to evaluate associations between molecular subtype and established histopathological risk factors.

Quality control measures included external positive controls and internal tissue controls for IHC. Equivocal/discordant cases were repeated on deeper sections and/or alternate tumor blocks. For POLE sequencing, DNA quality and quantity were assessed prior to PCR amplification; sequencing was performed using Sanger methodology, and ambiguous results were repeated. The actual IHC non-evaluable rate in our study was 7%, and the actual POLE non-evaluable rate was 0%.

Statistical analysis

The collected data were organized in Microsoft Excel. Complete case analysis was performed. The data were analyzed using the chi-square test to assess associations between molecular markers and histopathological characteristics, utilizing IBM SPSS Statistics for Windows, Version 26.0, 2019 (IBM Corp., Armonk, NY). Given low numbers in rare molecular groups and low event rates, the study may be underpowered to detect small differences across some strata. A p-value of <0.05 was considered statistically significant.

## Results

Out of the 175 patients initially enrolled, one patient was found to have endometrial stromal sarcoma on final histopathology, and 14 patients had incomplete medical records. After excluding these 15 cases, a total of 160 patients were included in the study. The age at onset ranged from 30 to 87 years, with a mean age of 61.9 years. The majority of patients underwent minimally invasive surgery, while only 16 had open surgery. Out of 160, 129 underwent robotic-assisted surgery (80.62%), and 15 patients had laparoscopic surgery (9.37%). Table 1 summarizes the histopathological and molecular parameters associated with tumor stage, grade, and histologic type.

**Table 1:** Histopathological and Molecular Parameters Associated With Tumor Stage, Grade, and Histologic Type (N=160) *Significant value MMR-d: mismatch repair deficiency; NSMP: No specific molecular profile

Histopathological parameters	Number of patients	POLE-mt	MMR-d	p53-abn	NSMP	Chi-square	Degree of freedom	P-value
Number of patients	160	1 (0.6%)	29 (18.1%)	33 (20.6%)	99 (61.9%)			-
Stage						9.733	6	0.136
Stage I	146 (91.3%)	1	26 (89.7%)	28 (84.8%)	93 (93.9%)			
Stage II	04 (2.5%)	0	2 (6.9%)	2 (6.1%)	0			
Stage III	10 (6.2%)	0	1 (3.4%)	3 (9.1%)	6 (6.1%)			
Grade						58.892	3	1.01 × 10⁻¹²^*^
Low	114 (71.3%)	0	27 (93.1%)	6 (18.2%)	81 (81.8%)			
High	46 (28.7%)	1	2 (6.9%)	27 (81.8%)	18 (18.2%)			
Histological type						62.071	3	<0.0001^*^
Endometrioid	126 (78.7%)	0	28 (96.6%)	10 (30.3%)	89 (89.9%)			
Non-endometrioid	34 (21.3%)	1	1 (3.4%)	23 (69.7%)	10 (10.1%)			

The majority of patients (91.3%) presented with Stage I disease. Endometrioid carcinoma was the most common histological subtype (78.7%), followed by serous (12.5%), clear cell (3.1%), mixed Müllerian tumor (MMT) (3.75%), mucinous (1.2%), and other subtypes. Low-grade tumors (Grade 1 and 2 endometrioids) accounted for 71.3% of cases, while high-grade tumors (Grade 3 endometrioid and non-endometrioid histologies) comprised 28.7%.

Histopathological parameters and molecular markers associated with myometrial invasion and LVSI are presented in Table 2.

**Table 2:** Histopathological Parameters and Molecular Markers Associated with Myometrial Invasion and LVSI (N=160) *Significant value MMR-d: mismatch repair deficiency; NSMP: No specific molecular profile; LVSI: Lymphovascular space invasion

Histopathological parameters	Number of patients	POLE-mt	MMR-d	p53-abn	NSMP	Chi-square	Degree of freedom	P-value
Number of patients	160	1 (0.6%)	29 (18.1%)	33 (20.6%)	99 (61.9%)			-
Myometrial invasion						7.671	6	0.263
No	23 (14.4%)	1	4 (13.8%)	6 (18.2%)	13 (13.1%)			
<50%	74 (46.2%)	0	16 (55.2%)	13 (39.4%)	46 (46.5%)			
>50%	63 (39.4%)	0	9 (31%)	14 (42.4%)	40 (40.4%)			
LVSI						9.872	3	0.020^*^
No/Focal	147 (91.9%)	1	28 (96.6%)	26 (78.8%)	94 (94.95%)			
Substantial	13 (8.1%)	0	1 (3.4%)	7 (21.2%)	5 (5.05%)			
Lymph node status						1.699	6	0.945
Not identified	8 (5%)	0	2 (6.9%)	2 (6.1%)	5 (5.05%)			
Not involved	141 (88.1%)	1	24 (82.8%)	28 (84.8%)	89 (89.9%)			
Nodal metastasis	11 (6.9%)	0	3 (10.3%)	3 (9.1%)	5 (5.05%)			

Myometrial invasion of less than 50% was observed in 46.2% of patients, while 50% or more invasion was present in 39.4%. Substantial LVSI, defined as involvement of five or more vessels, was noted in 8.1% of patients. Nodal metastasis was observed in 6.9% of cases, whereas lymph nodes were not identified in 5% of the cohort. A total of 162 mutations were identified among 160 patients; one patient exhibited concurrent p53 mutations and POLE mutations, while another demonstrated co-existing p53 mutations and MMR deficiency. POLE mutations were identified in 0.6% of cases (1 out of 160 patients). MMR-d was observed in 18.1% of patients, while 81.9% were MMR proficient. p53 mutations were detected in 20.6% of patients, with 79.4% showing wild-type p53 expression. The NSMP group represented the largest molecular subgroup, comprising 61.9% of the cohort. The single POLE-mt case was a Stage I, Grade 1 tumor with MMT histology. This patient exhibited no myometrial invasion, LVSI, or nodal involvement. Among MMR-d cases, the majority (96.6%) were associated with endometrioid histology, with one case identified as dedifferentiated carcinoma (endometrioid + undifferentiated). MMR-d was predominantly seen in low-grade tumors (93.1%) with varying degrees of myometrial invasion. LVSI was present in 1 out of 29 MMR-d cases, and nodal metastasis was observed in 3 cases. p53-abn tumors were more commonly associated with high-grade tumors (81.8%), non-endometrioid histology (69.7%), myometrial invasion (81.8%), and substantial LVSI in 21.2% of cases. Among NSMP cases, only 10 out of 99 were associated with non-endometrioid histology, and 6 were classified as Grade 3 endometrioid tumors.

**Figure 2:**
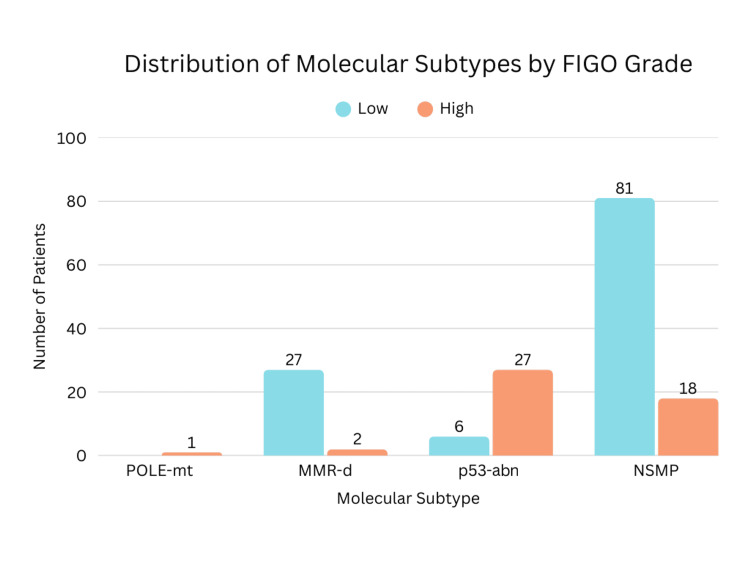
Distribution of Molecular Subtypes According to Tumor Grade

**Figure 3:**
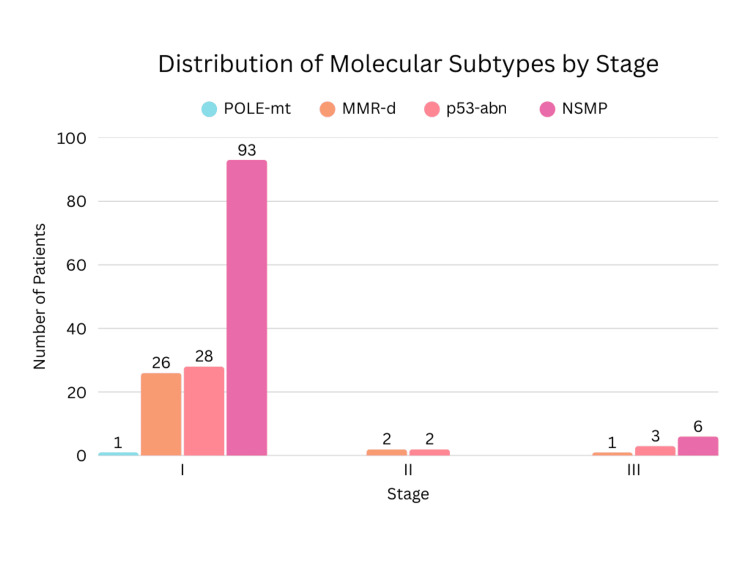
Distribution of Molecular Subtypes According to Stage of Disease

**Figure 4:**
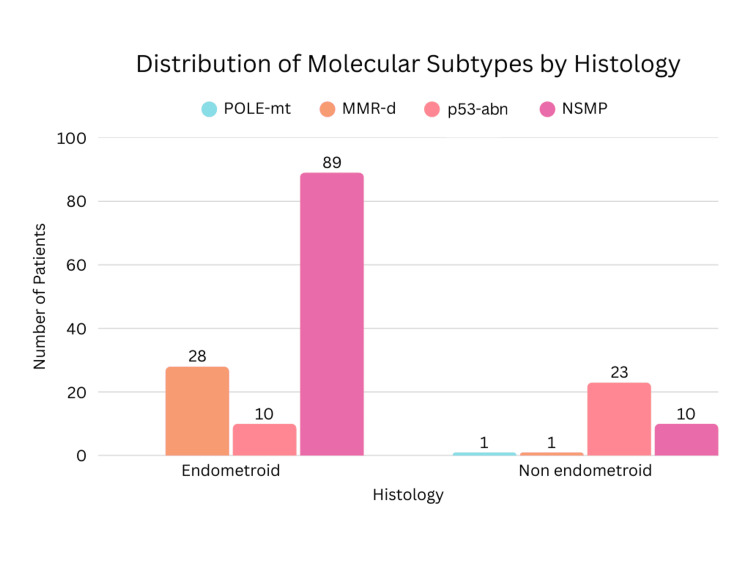
Distribution of Molecular Subtypes According to Histologic Type

Myometrial invasion was seen in 86.9% of NSMP cases. Substantial LVSI and nodal involvement were observed in 5 out of 99 patients. Nodal metastasis was observed in p53, MMR-d, and NSMP subtypes. One patient (0.62%) exhibited both POLE mutation and p53 abnormality and was classified and managed as POLE-mt, given its favorable prognosis. Similarly, one patient demonstrated both p53 abnormality and MMR deficiency and was categorized under the MMR-d, following the hierarchical classification approach. Statistically significant associations were observed between molecular subtypes and histological type (p<0.0001) as well as LVSI (p=0.020). Tumor grade demonstrated a highly significant association with molecular classification (p<0.0001). However, no statistically significant correlations were found between molecular subtypes and FIGO stage (p=0.136), depth of myometrial invasion (0.263), or lymph node involvement (p=0.945). The distribution of molecular subtypes according to tumor grade, disease stage, and histologic type is presented in Figures 2-4, respectively.

## Discussion

The integration of molecular markers into the classification of EC has proven valuable for both prognostication and guiding clinical management. While comprehensive genomic analysis remains costly, IHC for p53 and MMR proteins offers a cost-effective and practical alternative [5-6, 15-17]. A study comparing molecular subtyping of EC using NGS and the ProMisE algorithm reported a remarkably high concordance rate of 98.1%, with 52 out of 53 cases showing identical classification results (κ=0.97) [18]. The only discordant case involved a tumor that was microsatellite-stable on NGS but exhibited loss of MLH1 and PMS2 expression, highlighting the occasional complexity in interpreting MMR status across different platforms. This high level of agreement underscores the reliability and clinical applicability of the ProMisE classifier as a cost-effective and accessible alternative to more advanced molecular methods such as NGS, particularly in routine diagnostic settings. Moreover, ProMisE has been validated as a robust prognostic tool. It has demonstrated significant predictive value for both progression-free survival (PFS; p=0.001) and disease-specific survival (DSS; p=0.03), independent of conventional clinicopathologic risk factors [19]. This emphasizes its potential in refining risk stratification beyond traditional parameters like tumor grade and stage. In addition, the classifier shows strong reproducibility across tissue samples, with high concordance between diagnostic biopsies and surgical specimens, as evidenced by an accuracy of 0.91 and a kappa statistic of 0.88. These findings affirm the utility of ProMisE not only in prognostication but also in guiding individualized management strategies, offering a standardized approach to molecular classification in EC. As a result, the incorporation of molecular markers into risk stratification has become standard practice in the management of EC. In this study, we evaluated the association between molecular subtypes and various histopathological parameters using IHC-based methods. The findings are consistent with the molecular classifications proposed by TCGA and validated by the ProMisE model.

In our study, MMR deficiency was identified in 18.1% of cases, indicating a moderate prevalence of this molecular subtype within our cohort. This rate is notably lower than that reported in the final validation of the ProMisE classifier, which evaluated a large, population-based series and found MMR-d in 28.1% of cases [19]. The discrepancy between our findings and those of the larger validation study may be attributed to differences in patient demographics, tumor characteristics, or geographic and ethnic variations. Despite the lower prevalence observed in our cohort, MMR-d remains a clinically relevant subgroup due to its association with an intermediate prognosis and potential responsiveness to immunotherapy. These findings emphasize the need for comprehensive molecular profiling in EC to ensure accurate classification and personalized treatment planning. The MMR-d tumors in our cohort were predominantly associated with low-grade histology (93.1%), consistent with their hypermutated phenotype and intermediate prognosis. This finding aligns with previous studies, including that of Favier et al., which reported MMR deficiency in up to 30% of endometrioid carcinomas [20,21]. Another study by Huvila et al. [18] reported a notably higher prevalence of MMR-d, identifying it in up to 43.5% of EC cases. This elevated frequency further underscores the variability in MMR-d prevalence across different populations and study designs. The strong association between MMR protein expression and tumor histological grade has also been well documented. Serin et al. [22] demonstrated a correlation between MMR deficiency and higher histological grades, suggesting that loss of MMR function may contribute to tumor progression and dedifferentiation. In support of these observations, Eriksson et al. [23] found that a majority of MMR-d EC cases, 82.2%, were diagnosed at an early stage (Stage I), and 73.7% of these tumors were classified as low-grade. These findings highlight a paradox often seen with MMR-d tumors: despite their higher mutational burden and occasional association with adverse histological features, they frequently present at an early stage and with favorable tumor grade, contributing to their relatively good prognosis.

This complex biological behavior reflects the need to integrate both molecular and histopathological parameters in order to guide clinical decision-making in EC. Additionally, we observed that myometrial invasion was common among MMR-d cases (86.2%), with nodal involvement present in 10.3% of these patients. MMR deficiency is associated with microsatellite instability (MSI), a hallmark of Lynch syndrome, a hereditary cancer syndrome that predisposes individuals to various malignancies, including EC [24]. However, in sporadic cases of EC, MMR deficiency may also arise through somatic mutations or epigenetic silencing of MMR genes [25]. In the study conducted by Eriksson et al. [23], 13.6% of EC patients with MMR-d experienced disease recurrence or progression during the follow-up period. Despite this moderate rate of recurrence, the MMR-d group demonstrated a favorable long-term outcome, with a reported five-year overall survival rate of 90% [23]. These findings suggest that while MMR-d tumors may carry an intermediate risk of recurrence compared to other ProMisE subtypes, the overall prognosis remains relatively good. The high survival rate may be attributed to the typically early-stage presentation and endometrioid histology frequently observed in MMR-d tumors, as well as the potential for improved immunogenic response due to the high mutational burden associated with MMR deficiency. The identification of MMR deficiency using immunohistochemical staining has significant implications for the diagnosis, molecular classification, and clinical management of EC.

In this study, we also analyzed the IHC staining pattern of p53, a tumor suppressor gene frequently mutated in a variety of malignancies, including EC. These tumors correspond to the copy-number high subgroup in the TCGA classification and are associated with poor prognosis. In our cohort, p53-abn mutations were identified in 20.6% of cases, indicating a substantial representation of this high-risk molecular subtype. This finding is consistent with the study by Eriksson et al., who reported p53-abn mutations in 15.9% of cases, with a notable 77.3% of these tumors demonstrating a non-endometrioid histological subtype, a pattern often associated with more aggressive clinical behavior and poorer prognosis [23]. In contrast, Huvila et al. reported a much higher prevalence, identifying p53-abn mutations in 64% of EC cases that were neither POLE-mt nor MMR-d, suggesting that p53-abn may be more frequent in tumors lacking other defining molecular alterations [18]. Similarly, a study by Kommoss et al. reported a lower prevalence of 12.2% for p53-abn mutations in their cohort, which, although somewhat lower than the 20.6% observed in our study, still underscores the variability in the distribution of molecular subtypes across different patient populations and study designs [19]. These differences may reflect underlying population genetics, tumor biology, or differences in the selection criteria and diagnostic techniques employed across studies.

In our cohort, p53-abn mutations were predominantly observed in high-grade (Grade 3) tumors and non-endometrioid histologies. The aggressive nature of p53-abn tumors is reflected in their frequent association with deep myometrial invasion, substantial LVSI, and nodal metastasis. Specifically, p53 mutations were observed in 39.4% of serous carcinomas, 30.3% of endometrioid carcinomas, and 15.1% of MMT and clear cell carcinomas. These findings are consistent with those of Saharti and Altaf, who reported a higher frequency of p53 mutations in papillary serous (45.8%) and MMT subtypes [26]. Other studies have similarly shown p53 mutations in approximately 50% of papillary serous carcinomas and up to 80% of MMT cases [27,28]. The aggressive clinical behavior associated with p53-abn tumors was further substantiated by the findings of Eriksson et al. [23]. Their study demonstrated that patients with p53-abn EC exhibited the highest rates of both disease recurrence and cancer-specific mortality compared to other ProMisE molecular subtypes. Moreover, this group had the lowest five-year overall survival rate, underscoring the poor prognosis associated with this molecular profile [23]. These results reinforce the classification of p53-abn tumors as a high-risk subgroup within the ProMisE framework, highlighting the need for intensified surveillance and potentially more aggressive therapeutic strategies in managing patients with this subtype.

The observed MMR deficiency in a subset of tumors highlights underlying defects in DNA repair mechanisms. The strong association between MMR deficiency and specific histological subtypes, particularly endometrioid and papillary serous carcinomas, underscores the distinct molecular pathways involved in endometrial carcinogenesis. Additionally, the co-occurrence of p53 mutations in MMR-d tumors suggests a potential overlap between these genetic alterations, further supporting their combined role in tumor progression. Some studies have reported a higher prevalence of advanced-stage disease in cases harboring both p53 mutations and MMR deficiency [29]. In contrast, our study identified a single patient with concurrent p53 mutation and MMR deficiency who presented with endometrioid histology, Stage I disease, Grade 2 tumor, less than 50% myometrial invasion, and no evidence of LVSI or lymph node involvement. These findings emphasize the importance of evaluating both p53 and MMR status in all patients with EC, as their combined assessment can enhance risk stratification and inform prognostic evaluation more accurately than either marker alone. In contrast to the 13% prevalence of POLE mutations reported by Huvila et al. [18], our cohort demonstrated a markedly lower incidence, with only one patient (0.6%) identified as harboring a POLE mutation. This significant discrepancy highlights potential differences in population genetics, sample size, or methodological variations between studies. Similarly, another study reported POLE mutations in 9.3% of EC cases, which, while lower than Huvila’s findings, still represents a substantially higher frequency compared to our dataset [19]. These findings suggest that the distribution of ProMisE molecular subtypes, particularly POLE-mt, may vary considerably across different geographic regions and patient populations, underlining the importance of population-specific molecular profiling in EC.

Similar to the findings reported by Van Gool et al., we observed that POLE-mt tumors, though rare in our cohort, were confined to Stage I disease [30]. A systematic review and meta-analysis evaluating the clinical characteristics of the ProMisE molecular subgroups of EC reported that 93.7% of patients classified under the POLE-mt group were diagnosed at stage I, indicating a strong association between this molecular subtype and early-stage disease at presentation [31]. Consistent with these findings, Eriksson et al. observed that 88.5% of patients in the POLE-mt group were diagnosed at stage I. This group was also associated with the most favorable clinical outcomes, including the lowest rates of recurrence and disease-specific mortality. Furthermore, patients with POLE-mt tumors exhibited the highest 5-year overall survival rate among all ProMisE subtypes, reinforcing the prognostic significance of POLE mutations and their role in guiding risk stratification and treatment decisions in EC [23]. In our study, the POLE mutation co-occurred with p53 mutation in a case of MMT histology, which demonstrated no myometrial invasion, no LVSI, and no lymph node involvement. Nodal metastasis and LVSI were predominantly observed in p53-abn and MMR-d tumors, consistent with previous findings [29]. However, in our study, no significant correlation was found between molecular subtypes and FIGO stage, myometrial invasion, or nodal status. This may be attributed to the predominance of early-stage disease within our study cohort. These observations underscore the added value of molecular classification, highlighting its utility in prognostication and risk stratification beyond traditional histopathological staging parameters.

Our findings are consistent with existing literature and demonstrate the feasibility of implementing molecular classification through IHC and limited gene sequencing in routine clinical practice. Additionally, this study highlights the significant association between histopathological parameters and molecular subtypes. It underscores the role of molecular markers in differentiating histological subtypes of EC and their value in providing prognostic insight. This integrated approach can contribute meaningfully to refining risk stratification and guiding personalized management strategies for patients with EC.

Limitations

This study has several limitations. The single-center retrospective design and relatively small sample size may limit generalizability and introduce potential selection bias, although the single-center setting ensured uniform surgical-pathological protocols and standardized molecular testing. The low numbers in certain molecular subgroups and low event rates may have limited the power to detect small differences across strata. Additionally, the predominance of early-stage endometrial cancer in the cohort may have influenced correlations between molecular subtypes and clinicopathological features. Another limitation is the lack of comprehensive prognostic data, including long-term survival outcomes and details of adjuvant therapies, which were not systematically available for all patients. Larger prospective, multi-center studies with longer follow-up are needed to further validate these findings and better establish their prognostic significance.

## Conclusions

This study reinforces the clinical relevance of integrating molecular classification with conventional histopathological assessment in EC. Using an IHC-based ProMisE framework supplemented by targeted POLE sequencing, we demonstrated significant associations between molecular subtypes and key histopathological parameters, particularly tumor grade, histologic type, and LVSI. p53-abn tumors were strongly associated with high-grade, non-endometrioid histology and adverse pathologic features, while MMR-d tumors predominantly exhibited low-grade endometrioid morphology with intermediate-risk characteristics. Although POLE mutations were rare in our cohort, consistent with geographic variability, the identified case demonstrated favorable pathological features in line with previously reported excellent prognosis. Importantly, no significant association was observed between molecular subtype and FIGO stage or depth of myometrial invasion, likely reflecting the predominance of early-stage disease in this cohort. These findings underscore that molecular profiling provides prognostic stratification beyond traditional staging alone. Integrating cost-effective, IHC-based molecular classification into routine practice enhances risk stratification and prognostic accuracy, supporting personalized management in EC and advancing precision oncology in real-world settings.
